# Animal models of pyruvate dehydrogenase complex deficiency: insight into mechanisms of cerebral abnormalities and tissue-specific role in metabolism

**DOI:** 10.3389/fmed.2026.1861016

**Published:** 2026-06-22

**Authors:** Mulchand S. Patel, Todd C. Rideout

**Affiliations:** 1 Department of Biochemistry, Jacobs School of Medicine and Biomedical Sciences, University at Buffalo, The State University of New York, Buffalo, NY, United States; 2 Department of Exercise and Nutritional Sciences, School of Public Health and Health Professions, University at Buffalo, The State University of New York, Buffalo, NY, United States

**Keywords:** animal models, cerebral development abnormalities, high-fat diet, murine, phenylbutyrate, pyruvate dehydrogenase complex deficiency, tissue-specific metabolic roles, zebrafish

## Abstract

Primary pyruvate dehydrogenase complex (PDC) deficiency results from inborn errors in the genes encoding its component proteins with largely devastating outcomes. Among its genes, the X-linked *PDHA1* gene is subject to a much higher rate of mutations. Despite the analysis of many PDC-deficient subjects, no specific genotype–phenotype relationship emerges from the available data. This review focuses on the observations from animal models of primary PDC deficiency. Mouse models of systemic and brain-specific PDC deficiency closely reproduced several cerebral abnormalities observed in many PDC-deficient subjects and provide new insights into the impairment of cellular proliferation, migration and differentiation. Mouse models would be useful tools to evaluate efficacy of dietary and drug treatments. The mouse model is useful for creating tissue-specific PDC deficiency to examine the importance of PDC in carbohydrate metabolism. Other animal models of PDC deficiency have provided unique insights on the impact of PDC deficiency and are useful tools for rapid screening of drugs. All animal models utilized so far carried null mutations in the PDC genes, and hence creations of missense mutations in animals, especially in the mouse, are highly desirable to evaluate the genotype–phenotype relationship in PDC deficiency.

## Introduction

**Figure 1:**
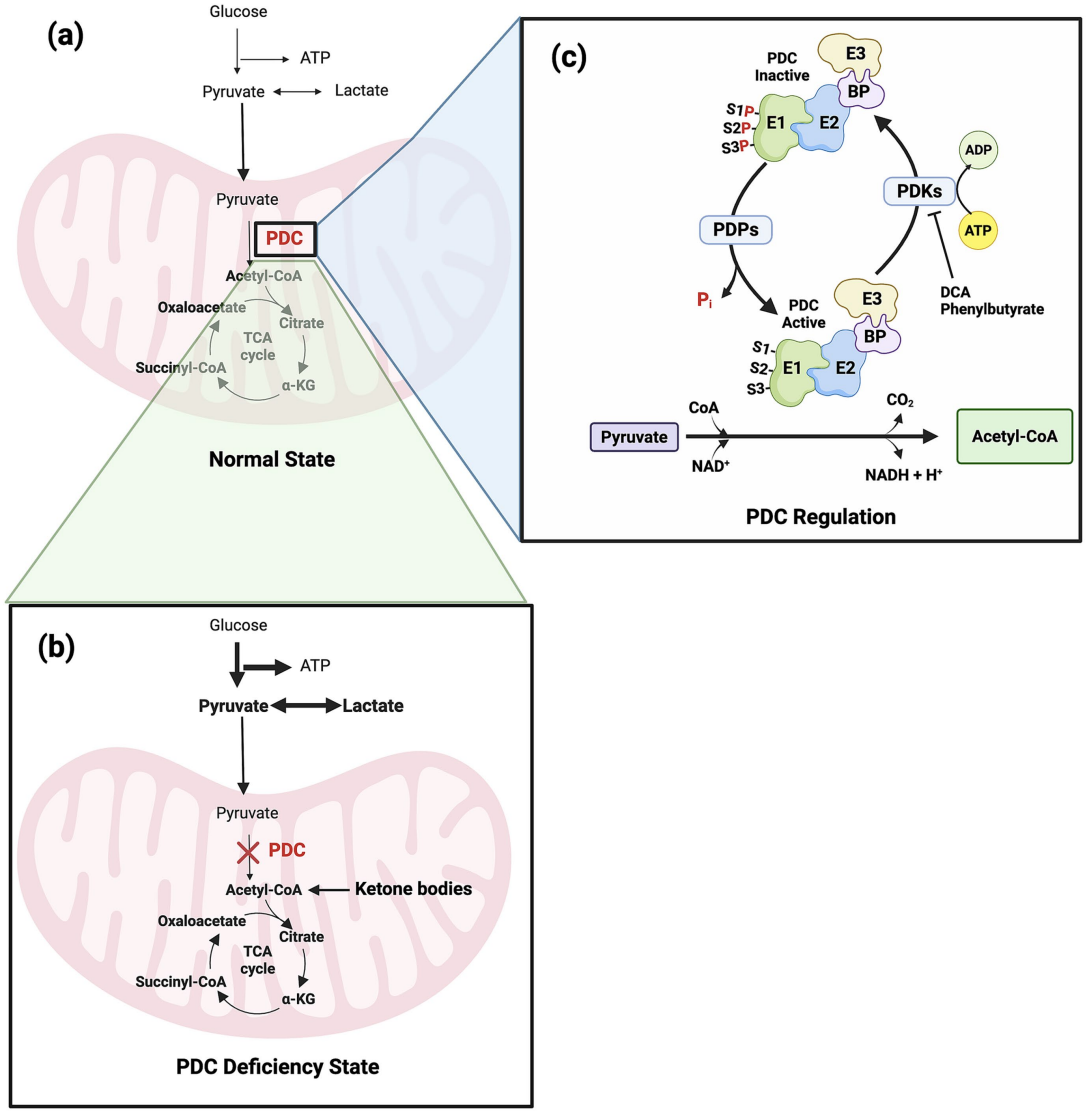
Schematic representation of glucose-carbon through PDC in the brain during normal **(a)** and PDC-deficient **(b)** states, and PDC regulation by a phosphorylation/dephosphorylation mechanism **(c)**. DCA, dichloroacetate; *α*-KG, α-ketoglutarate; TCA cycle, tricarboxylic acid cycle.

Pyruvate dehydrogenase complex, (PDC), a multienzyme complex, catalyzes the irreversible oxidative decarboxylation of pyruvate to acetyl-CoA in the mitochondria, and is the primary link between glycolysis and the tricarboxylic acid cycle for glucose metabolism. PDC is comprised of three catalytic components: pyruvate dehydrogenase (PDH or E1), dihydrolipoamide acetyl-transferase (DLAT, E2) and dihydrolipoamide dehydrogenase (DLD, E3), and a non-catalytic component referred to as E3-binding protein (E3BP; formerly known as protein X; Figure 1). The PDH (E1) component is a heterotetramer composed of two *α* and two *β* subunits (1, 2). In mammals, there are two isoforms of the E1α encoded by two different genes: (i) the X-linked gene (*PDHA1* in humans and *Pdha1* in mice) codes for E1α protein in all somatic cells, and (ii) an autosomal, intronless gene (*PDHA2* in humans and *Pdha2* in mice) encodes the variant E1α in the testis (3, 4). Mammalian PDC is post-translationally regulated by a family of PDH kinases (PDK 1–4 isoenzymes) and PDH phosphatases (PDPs 1–2) (5, 6). PDKs phosphorylate three specific serine residues in the E1α subunit, causing inactivation of PDH and hence PDC whereas dephosphorylation of PDH by PDPs activates of PDH and hence PDC (Figure 1).

Genetic defects in the human genes encoding catalytic and regulatory proteins as well as non-catalytic binding protein have been reported, and their occurrences vary markedly (Table 1). Approximately 80% of PDC deficiency cases result from mutations in the X-linked *PDHA1* gene, and about 140 pathological variants have been reported in the *PDHA1* gene. PDC has an incidence of about 50,000–75,000 live births/year in North America (7). It was estimated that approximately 2000 cases of PDC deficiency existed in the United States in 2025, however, only about one half of them are diagnostically confirmed. The clinical phenotypic spectrum is heterogeneous and not distinctive and includes as major findings: congenital lactic acidosis with neonatal death, intrauterine growth retardation, developmental delay, primary or acquired microcephaly, hypotonia, cerebral atrophy, dysgenesis or agenesis of the corpus callosum and occasional occurrence of epilepsy, hypertonia and ataxia (8–12). Correlations between specific genotypes and phenotypes have not been established due to variability in clinical presentations, delay in establishment of diagnosis, and variations in the start as well as the degree of dietary carbohydrate restriction for PDC patients with the same identified mutations (8, 13). However, animal models harboring specific molecular or tissue types of PDC deficiency may be useful in elucidating important genotype–phenotype correlations.

**Table 1:** Percentage distribution of PDC gene-specific mutations in humans.

PDC enzyme component (Abbreviations)	Gene	Chromosome	% of all PDC deficiency cases^a^
Pyruvate Dehydrogenase (PDH, E1)	*PDHA1*	X	76–85
	*PDHA2*	4	-
	*PDHB*	3	4–9
Dihydrolipoamide acetyltransferase (DLAT, E2)	*DLAT*	11	1–4
Dihydrolipoamide dehydrogenase (DLD, E3)	*DLD*	7	1–6
E3-binding protein (E3BP)	*PDHX*	11	7–11
Pyruvate dehydrogenase kinase (PDK1-PDK4)	*PDK1*	2	-
	*PDK2*	17	-
	*PDK3*	X	<1
	*PDK4*	7	-
Pyruvate dehydrogenase phosphatase (PDP1, PDP2)	*PDP1*	8	~1
	*PDP2*	10	-

^a^This information is taken from a review by Ganetzky et al. (67).

The aims of this review are two-fold: (i) to emphasize the utility of animal models in defining PDC’s role on energy metabolism and possible genotype–phenotype correlations under well-defined experimental conditions and (ii) to evaluate the potential beneficial effects of dietary modifications and drug treatments to enhance the activity of the residual mutated PDC.

## Animal models for PDC deficiency

In this review we will include animal models only; although the use of modified cultured cells has provided interesting information about PDC deficiency on cellular processes, they do not serve as models for the impact of PDC deficiency in intact organisms and are not included. Furthermore, primary PDC deficiency can develop from mutations in one of its catalytic component proteins, the binding protein or regulatory proteins, whereas secondary PDC deficiency can develop from mutations in the synthesis and activation of its requisite cofactors, such as thiamine and lipoic acid, or indirectly from mutations in several other mitochondrial proteins. Here, we will review the animal models for primary PDC deficiency only. Since mutations in the X-linked *PDHA1* gene represent the predominant cause of PDC deficiency (Table 1), the random inactivation of one of the two X chromosomes in female patients has created a condition in which the brain is severely affected exhibiting gross cerebral changes with elevated concentrations of pyruvate and lactate in cerebrospinal fluid (14, 15). Although these patients appeared to have generalized defects in PDC activity as detected in cultured fibroblasts, blood pyruvate and lactate levels were only slightly raised without causing systemic acidosis in these patients. The condition in these patients was termed as ‘cerebral’ lactic acidosis due predominantly to brain-specific PDC deficiency. Mouse models have provided an opportunity to evaluate the effects of brain-specific PDC deficiency on cerebral development and function without any systemic contributions, and hence this condition is also presented below with systemic PDC deficiency.

### Mouse models of PDH deficiency

#### Systemic PDH deficiency

A murine model of PDC deficiency was developed by introducing a silent mutation into the mouse X-linked *Pdha1* gene by inserting two loxP sequences into intronic sequences flanking exon 8 to create the *Pdha1^flox8^* allele which can be cleaved to delete exon 8 by expressing the transgene of Cre recombinase (16). This murine line is widely used for investigating PDH deficiency under systemic or cell-specific conditions, as summarized below (Tables 2, 3). When homozygous *Pdha1^flox8^* females were bred with male mice harboring a transgene *EIIa-Cre,* there was a delayed development of some embryos by 9.5 days postcoitus followed by resorption after a couple of days (Table 2) (16). Furthermore, no male offspring were born, resulting in a reduction in the average litter size (17). Brain weights of PDH-deficient females were reduced and PDC activity was reduced in the brain and other tissues. Glucose oxidation and lipogenesis from glucose by the brain were markedly reduced. Histological and immunodetection analyses revealed reductions in structures in white and gray matters, including the thickness of the neocortex and the granular layer of cerebellar cortex, as well as size reduction of the corpus callosum, and in the fibers of the striatum, reticular thalamic nucleus, cerebellar fissures, and the pyramids (17). These findings are similar to those observed in the brains from PDC-deficient patients (8–12). Furthermore, the densities of granular neurons and Purkinje neurons were decreased and dendritic arbor development in Purkinje neurons was impaired. The proliferation, migration and differentiation into neurons by newly generated cells were reduced in PDH-deficient females during pre- and postnatal periods (17).

**Table 2:** Summary of animal models with mutated PDC genes.

Mutated gene	Model (Species)	Breeding and tissues (cells) affected	Key findings and treatments	References
*Pdha1*	Mouse	Flox8-females vs. transgenic males (*EIIa-Cre*); Systemic PDC deficiency	Creation of a Flox8 mouse colony; delayed development of some embryos by 9.5 days postcoitus followed by resorption by day 11.5.	(16)
*Pdha1*	Mouse	Flox8-females vs. transgenic males (*EIIa-Cre*); Systemic PDC deficiency	Only female progeny was born; PDC deficiency in tissues; ~ 25% reduction in the brain; impairment in brain structures development; impaired cell development, migration and differentiation into granular and Purkinje neurons.	(17)
*Pdha1*	Mouse	Flox8-females vs. transgenic males (*Nes-Cre*); Mild PDC deficiency (~25%) in the brain only.	No male progeny born; detection of PDC-negative cells; cerebral structure abnormalities. Treatment: Beneficial effects on progeny brain weight and structural development when mothers were fed a ketogenic diet during gestation and lactation. Increased lactate level and loss of N-acetyl-aspartate and glutamine plus glutamate in the brain. Reduction in the Purkinje cell density with increased cell size. Treatment: Beneficial effects of phenylbutyrate on Purkinje cells and cells in SVZ, SGZ and the cortex with restored proliferation oligodendrocytes precursor cells. The relative fluxes of specifically labeled acetate and glucose were not altered in the matured brain of mildly PDC-deficient female mice.	(19, 22, 28–30)
*Pdha1*	Mouse	Flox8-females vs. transgenic males (*hGFAP-Cre*) PDH deficiency in neurons and astrocytes.	Male progeny was born but proved lethal by day 28; severe PDC deficiency (~50%) in neurons and astrocytes; presence of microencephaly and cortical atrophy; impaired glucose metabolism; reduction in in vivo cortical activation which was responsive to acetate metabolism. Treatment: Propionate treatment in conjunction with a ketogenic diet extended the lifespan, decreased neuronal loss and reactive astrocytosis, and some improvement in the motor phenotype in PDC-deficient male mice.	(23, 24)
*Pdha1*	Mouse	Flox8-females vs. transgenic males (Cnp*-Cre/+)* Or (PO-Cre) Or (*NG2-CreER*)	Axonopathy was specific to CnpCre/+ positive (oligodendrocytes and Schwann cells) cells in PDH-deficient male mice; no axonal pathology or myelin structures abnormality in PO-Cre (Schwann cells) or NG2-CreER positive (oligodendrocyte precursor cells) cells.	(25)
*Pdha1*	Mouse	~2.0 cM DNA deletion in X Chromosome (containing the *Pdha1* gene)	Deletion of the Pdha1 gene resulting in embryonic lethality of male embryos and development of neonatal lactic acidosis.	(26)
*Pdha1*	Rat	Delivery of viral (*scAAV8-si3-PDHA1*) vector in the right striatum and substantia nigra of rat brain	Reduction in the E1α and E1β proteins and in PDC activity after 14–25 weeks; abnormal contralateral rotation activity during a 90-min period after amphetamine injection.	(27)
*Pdhb*	Zebrafish	*Noir* mutant	Exhibiting vision defect-related darker appearance due to affected cholinergic amacrine cells of the inner retina; Treatment: a ketogenic diet partially rescued vision and prolonged survival of the larvae.	(35)
*dPdhb*	*Drosophila melanogaster*	Mutated *dPdhb* Neuron-specific deletion	Locomotor defects due to abnormal morphology of the motor neuron terminals at neuromuscular junctions; brain mitochondrial fragmentation; a rough eye phenotype; shorten the lifespan of adult flies.	(36)
*Dlat*	Zebrafish	noam631 zebrafish mutant with a missense mutation at codon 644 in the *Dlat* gene	Mutant fish had expanded melanophores, absence of feeding behavior, lethargy, and premature death and elevated levels of lactate. Treatment: Marked improvement in all these parameters in phenylbutyrate-treated fish.	(37–39)
*Dld*	Mouse	*Dld* gene disruption	Homozygous embryos died in utero; heterozygous female mice had about 50% reduction in activities of E3 as well as all E3-requiring complexes. Brain mitochondria from heterozygous DLD-deficient mice produced less H2O2 which was attributed to a greater loss of α-ketoglutarate dehydrogenase complex activity. Treatment: Injection of TAT-DLD fusion protein into heterozygous DLD-deficient mice resulted in increases in the activities of E3 and PDC in liver, heart and brain.	(40–42)
*Dld*	*C. elegans*	Feeding dld1-(RNAi) to worms	Increased levels of pyruvate, and reduced levels of ATP, survival, adult length and increased adaptive mitochondrial proliferation. Treatment: Dichloroacetate and thiamine singly or in combination were beneficial.	(34)
*Dld*	Zebrafish	Generation of the dldhcri3 model using CRIPSR/Cas9 technology	Shorten larval survival, reduced swim activity, hepatomegaly, and fatty liver; increased levels of lactate and branched-chain amino acids, and gross enlargement of mitochondrial structures. Treatment: Probucol or thiamine improved larval swim activity.	(43)
*Pdp1*	Canine	The spontaneous canine model of null mutation in the *Pdp1* gene.	PDP1-deficient dogs show minimal activation of PDC and increased blood lactate levels and exercise intolerance. Treatment: Dietary supplements of L-carnitine and high-fat diet are beneficial.	(44)

**Table 3:** Summary of tissue-specific PDC gene deletion in mice.

Mutated gene	Model (Species)	Breeding and tissues (cells) affected	Key findings and treatments	Refs
*Pdha1*	Mouse	Flox^8 -^females vs. transgenic males (*alb-Cre*) Liver-specific severe PDC deficiency	No lethality of male embryos; no incorporation of labeled glucose-carbon in fatty acids; increased lipogenesis in white adipose tissue (WAT); down regulation of lipogenic genes expression in the liver and upregulation in WAT; upregulation of expression of key glycolytic genes in the liver. PDC deficiency had no effect on proliferative capacity of normal hepatocytes and had modest effect on altered growth of aggressive hepatoblastoma.	(45–48)
*Pdha1*	Mouse	Flox^8 -^females vs. transgenic males (*Ins-Cre*) *β*-cell-specific PDC deficiency in pancreatic islets. Flox^8−^ females vs. transgenic males (*Ins-Cre*), tamoxifen injection	Reduced pancreatic insulin content and plasma insulin levels in males from the neonatal period onward; impaired insulin secretion capacity in adulthood; altered pancreatic islet morphology and gene expression capacity affecting β-cell mass and plasticity. Deletion of the *Pdha1* gene in β-cells in adulthood impaired glucose-stimulated insulin secretion and islets were enlarged with clear internal vacuoles.	(49–51)
*Pdha1*	Mouse	Flox^8 -^females vs. transgenic males (*Mck-Cre*) Heart/skeletal muscle-specific PDC deficiency.	PDC-deficient male progeny grew normally during the preweaning period but died ~7 days after weaning onto a standard rodent diet but survived on a high-fat diet; very low PDC activity (~5%) in hearts; developed left ventricular hypertrophy with increased myocyte diameter and reduced systolic function.	(52)
*Pdha1*	Mouse	Flox^8−^females vs. transgenic males (*α-MHC-CreER^T2^)* Heart-specific *Pdha1* deletion in adult mice by Tamoxifen treatment.	High mortality rate; larger myocardial infarcts size and macrophage infiltration in the hearts with increased hypertrophy and fibrosis; impairment in glucose oxidation in the perfused heart during ischemia/reperfusion; **Treatment:** No effect of dichloroacetate on hearts from PDH-deficient mice.	(54, 55)
*Pdha1*	Mouse	Flox^8−^females vs. transgenic males (*iHSA-Cre*) Skeletal muscle- specific *Pdha1* deletion after Tamoxifen treatment.	No impact on energy production, muscle contractile function or low intensity exercise performance in affected adult male mice but high intensity exercise performance severely impaired; **Treatment:** consumption of a high-fat diet had no effect on muscle insulin sensitivity in affected mice.	(53)
*Pdk2*	Mouse	Disruption of the *Pdk2* gene; systemic Pdk2 deficiency; skeletal muscle activity investigated.	PDK2 deficiency resulted in lower PDH activation and lower total PDC activity in muscle; compensatory increase in PDK1 activity for the lack of PDK2. In the fed state, PDK2 deficiency resulted in higher PDC activity and lower blood glucose levels but had no effect in the fasted state. PDK4 deficiency caused similar effects only after fasting. Double deficiency intensified these effects in both the fed and fasting states. PDK2 activity increases during osteoclast differentiation. PDK2 deficiency delayed bone loss and decreased the number of osteoclasts in ovariectomized mice.	(56, 57, 60)
*Pdk4*	Mouse	Disruption of the *Pdk4* gene; systemic deficiency; several tissues examined.	Starved PDK4-deficient mice had: (i) greater reduction in blood glucose levels, (ii) higher PDC activity in most tissues except the liver, and (iii) reduced gluconeogenesis. Starved obese PDK4-deficient mice had lower blood glucose levels; slight improvement in glucose tolerance test and slight increased insulin sensitivity.	(58, 59)
*Pdk3*	*C. elegans*	CRIPSR-Cas9 mediated knock-in a missense mutation at amino acid residue 159 (R159H) in the *Pdk2* gene in (pdkh2R159H), and (ii) human wild-type (hPDK3WT) and (iii) mutant (hPDK3R158H) forms of PDK3 selectively expressed in GABAergic motor neurons.	Decreased body width, and ATP levels; increased susceptibility to oxidative stress; and impairment in axon-associated synaptic transmission; mutant worms overexpressing human WT and p. R158H forms of PDK3 in the GABAergic motor neurons exhibited progressive neurodegeneration.	(62)
*E4F1*	Mouse	Striated skeletal muscle-specific E4F1*^KO^* mice with DLAT deficiency	18-Month-old E4F1*^KO^* mice developed hypercontracted fibers and centralized regenerative fibers in striated skeletal muscle; decreased levels of mRNA and protein of *Dlat* causing reduction in PDC activity; developed lactic acidosis and had a marked reduction in physical endurance. Feeding a ketogenic diet or administration of dichloroacetate to E4F1^KO^ mice improved lactic acidosis and physical endurance.	(63)
*E4F1*	Mouse	E4F1 conditional KO mice vs. transgenic mice (*Nes-Cre)*, the central nervous system (CNS)-specific deletion of E4F1 with DLAT deficiency	Perinatal death of E4F1^(Nes)KO^; degenerated areas in forebrain, midbrain and hindbrain neuroepithelium in E16.5 E4F1^(Nes)KO^ embryos; the structural lesions appeared at E14.5; decreased levels of mRNA and protein levels of the *Dlat* gene causing reduction in PDC activity and in acetyl-CoA levels, and increased circulating lactate levels in the mutant embryos.	(65)

#### Brain-specific PDH deficiency

To generate brain-specific-PDH deficiency in the progeny, *Pdha1^flox8^* females were bred with male mice harboring the transgene *Nes-Cre* (specific for neurons and glia) (18). Absence of live male progeny indicated prenatal lethality. Impairments in all other parameters, such as body weight, PDC activity, glucose metabolism, and histological abnormalities in the brain of brain-specific PDH-deficient females (18) were similar to those observed in the brain from systemic PDH-deficient females (Table 2) (17), indicating that the observed cerebral abnormalities were caused by brain PDC deficiency and little, if any, from the systemic contributions. An increase in lactate concentration and a marked loss of N-acetyl-aspartate and glutamine plus glutamate concentrations were observed in the brain (Table 2) (19), consistent with the findings in PDC-deficient patients (20, 21). Contrary to expected outcome, the relative flux of [1,2-^13^C]acetate and [1,6-^13^C]glucose into the citric acid cycle as measured from the distribution of ^13^C in glutamate and glutamine in the brain was not altered in mildly PDH-deficient adult mice; instead, there was preferential oxidation of [1,2-^13^C]acetate relative to [1,6-^13^C]glucose as judged from a different 13C-labeling pattern in glutamate compared to that of glutamine, indicating preservation of the neuron–glia metabolic compartmentation in PDH-deficient adult mice (Table 2) (22).

When male mice that expressed a different Cre-expressing transgene (*hGFAP-Cre*; specific for neurons and astrocytes) were bred with floxed^8^-females, PDH-deficient male progeny were born; however, this condition proved lethal by postnatal day 28. Significant findings from brain-specific PDH-deficient male mice (ages P16 to P25) included: (i) severe PDH deficiency (~50% reduction in PDC) in neurons and astrocytes; (ii) microencephaly, cortical atrophy, and hypoplasia of the corpus callosum; (iii) impaired glucose metabolism with increased alanine and decreased glutamate levels in whole brain samples; (iv) increased abundance of ^13^C from [U-^13^C]glucose in lactate and alanine and decreased abundance of ^13^C in glutamate; and (v) acetate-sparing glucose utilization by PDH-deficient brain, and (v) cellular metabolism and excitability responsive to alternate fueling by acetate (Table 2) (23). Increased propionate was converted to succinyl-CoA in glia and contributed to the *in vivo* imaging phenotype in PDH-deficient brain (24). Combining oral administration of propionate with ketogenic diet to deficient mice resulted in an increase in life span and a decrease in neuronal loss and reactive astrocytosis, with improvement in the motor phenotypes (Table 2) (24).

To investigate the importance of PDC in myelinating glia, floxed^8^-females were bred with transgenic males harboring *Cre* recombinase under the promoter of either (i) *Cnp*^Cre/+(^in oligodendrocytes and Schwann cells, (ii) *PO* (in Schwann cells), or (iii) *NG2-Cre^ER^* (in oligodendrocytes precursor cells). The major findings were: (i) reduction in fiber density and signs of axonal degeneration in sciatic and optic nerves from *Cnp^Cre/+^* PDH-deficient male mice from 6 months of age, and (ii) absence of axonal pathology or alterations in myelin structures or thickness in 10 month-old male mice expressing either *PO-Cre* expressing Schwann cells or *NG2-Cre^ER^* expressing oligodendrocyte precursor cells (Table 2) (25).

#### Large DNA deletion in X chromosome

A murine X-linked mutation with the deletion of ~2.0 cM of genetic material (referred to as Stripey) includes the growth factor-regulated protein kinase gene (*Rsk2*) and the pyruvate dehydrogenase *α* subunit (*Pdha1*) gene and is associated with Coffin-Lowry syndrome and neonatal lactic acidosis causing lethality of male embryos (Table 2) (26).

#### Adeno-virus-mediated brain PDH deficiency

*Pdha1* gene deletion in the right striatum and substantia nigra of rats was achieved by stereotaxic delivery of self-complimentary adeno-associated virus 8 carrying a small interfering RNA directed against the *Pdha1* gene (scAAV8-si3-PDHA1) (27). After 20 weeks the siRNA-injected rats revealed increased contralateral rotation during the first 10 min after amphetamine injection and reduction in the total rotation for a total of a 90-min period. Expression of E1α and E1β as well as PDC activity in striatum were decreased at weeks 14 and 25, respectively (Table 2).

#### Treatments for PDC deficiency in mice

Ketogenic diets: High fat diet results in increased availability of ketone bodies in circulation, and hence an alternate source of acetyl-CoA for the brain. A possible beneficial effect of feeding a ketogenic diet to *Pdha1^flox8^* mothers during pregnancy and lactation on brain development in their progeny with brain-specific PDH-deficiency was investigated (28). Brain weight was normalized, with improvement in brain structural deficits in the PDH-deficient progeny of mothers fed a ketogenic diet (67% calories from fat) compared to PDH-deficient progeny of chow-fed mothers. The ketogenic diet provided increased levels of alternate metabolic fuels such as ketone bodies for the developing brain of PDC-deficient mice (Table 2) (28).

Phenylbutyrate treatment: Phenylbutyrate and dichloroacetate are inhibitors of PDKs and thus inhibiting PDKs causing less phosphorylated and hence more ‘active’ residual PDH (Figure 1). The effect of phenylbutyrate administration (once a day intraperitoneally 250 mg/kg body weight) from P2 to P35 on the cerebellar Purkinje cells in brain-specific PDH-deficient female progeny was investigated by Klejbor et al. (29). Histological analyses of different regions of cerebellar cortex from deficient females receiving daily saline injection showed reduction in the Purkinje cell density and increased cell size of the individual Purkinje cell soma. Phenylbutyrate administration showed variable improvement on Purkinje cell populations in PDH-deficient female mice (Table 2). Adverse effects of PDC deficiency in these treated mice were examined on three populations of cells: O4 expressing oligodendrocytes (OLG), double stained Ki67^+^/O4^+^ immature proliferating OPCs, and overall numbers of the proliferating cells (Ki67^+^) in the brain regions (SVZ, SGZ and the cortex). A reduction in all cell populations was observed in analyzed brain regions in PDH-deficient mice. Administration of phenylbutyrate had a beneficial effect on O4 OLG, with significant increases in SVZ and SGZ. Interestingly, this treatment restored depleted OPCs in PDH-deficient mice to the level detected in control mice, indicating that loss of O4 OLG in brain from PDH-deficient mice possibly reflects changes in OPCs (Table 2) (30).

Dichloroacetate treatment; Dichloroacetate (DCA), an inhibitor of PDKs (Figure 1), was tested in limited cases of PDC deficiency to treat lactic acidosis (10, 31). Employing a specific genetic haplotype test (the human GSTz1/MAAI gene which encodes a bifunctional enzyme glutathione transferase zeta1/maleylacetoacetate isomerase) for ‘fast’ and ‘slow’ responders to dichloroacetate metabolism, Shroads et al. observed that relevant doses of dichloroacetate were well tolerated by healthy adults and children with mitochondrial diseases (32). Furthermore, this genetics-based dosing of dichloroacetate was found safe and well tolerated by cancer patients with recurrent brain tumors (33). No rodent studies with PDC deficiency are reported employing dichloroacetate treatment. Interestingly, dichloroacetate treatment to *Dld*-deficient Caenorhabditis elegans was found to be beneficial singularly or in combination with thiamine (Table 2) (34).

#### Pdhb deficiency

A zebrafish noir mutant (mutation in the *Pdhb* gene) exhibited a vision defect-related darker appearance due to affected cholinergic amacrine cells of the inner retina which was attributed to the inability to produce acetylcholine. Treatment with a ketogenic diet partially rescued vision and prolonged survival of the larvae (Table 2) (35). Neuron-specific mutation of *dPDHB* in *Drosophila melanogaster* shortened the lifespan of adult flies and induced locomotor impairment in larval and adult flies resulting from abnormal morphology of the motor neuron terminals at neuromuscular junctions. The mutation caused mitochondrial fragmentation in brains and developed a rough eye phenotype and aberrant photoreceptor axon targeting (Table 2) (36).

#### DLAT (E2) deficiency

The *noa^m631^* zebrafish mutant (harboring a missense mutation at codon 644 in the *DLAT* gene) had elevated levels of whole-body lactate and pyruvate and exhibited expanded melanophores, absence of feeding behavior, lethargy, and premature death (37, 38). When the mutant larvae were placed in a medium supplemented with a fatty acid emulsion, they displayed a marked improvement in their visual response resulting in improvement in feeding behavior. They also demonstrated reduced lactic acidosis and increased survival (38). In phenylbutyrate-treated fish, there was marked improvement in all these parameters, showing its potential use for treating PDC deficiency (Table 2) (39).

#### DLD (E3) deficiency

The mouse *Dld* gene was disrupted by the insertion of a neo gene into exon 10, resulting in the absence of detectable mRNA from the mutated allele (*Dld^tm1mjp^*) (40). Homozygous animals (*Dld^−/−^)* died prematurely with development delay at 7.5 postcoitum followed by resorption by 9.5-day postcoitum. Heterozygous mice (*Dld^+/−^)* were like wild-type littermates in gross appearance, behavior, and fertility. About 50% reduction in E3 activity with marked reductions in the activities of PDC and other E3-requiring complexes were observed in heterozygous mice (Table 2) (40).

Brain mitochondria isolated from heterozygous *Dld*-deficient mice (*Dld^+/−^)* produced significantly less H_2_O_2_ under conditions of maximum respiration than from mitochondria from littermate wild-type mice (41). This was attributed to a greater reduction in *α*-ketoglutarate dehydrogenase complex activity compared with other E3-requiring complexes. To achieve enzyme replacement therapy, the delivery by a single intravenous injection of TAT-DLD (the transcriptional activator of transcription-DLD) significantly increased E3 and PDC activities within liver, heart and brain for about 48 h (Table 2) (42).

DLD deficiency induced in Caenorhabditis elegans by feeding dld1(RNAi clone LLC1.3; which encodes a dsRNA against dld-1 when induced by isopropyl-*β*-D-thiogalactoside that reduces DLD-1 expression in *C. elegans*) resulted in increased levels of pyruvate and reductions in ATP levels, and decreased survival rate, adult length, and neuromuscular function (34). Mitochondrial unfolded protein stress response induction and adaptive mitochondrial proliferation were markedly increased in DLD-deficient worms. Dichloroacetate and thiamine supplementation showed individual and synergistic therapeutic benefits only in short-lived worms grown on 1:20 dld-1(RNAi) dilution bacteria and had no significant effect in normalizing the life span of the long-lived worms grown on full-dose dld-1(RNAi; Table 2). A zebrafish model of DLD deficiency, dld^cri3^, was developed by deleting 5 bp, causing the absence of the protein (43). Phenotypic analysis of DLD-deficient larvae revealed shorter survival, uninflated swim bladder, reduced swim activity, hepatomegaly and fatty liver. They had increased levels of pyruvate, lactate and branched-chain amino acids and their keto-acids. Mitochondria from liver, intestine and muscle showed gross enlargement with severe disruption of cristae and reduction in matrix electron density (Table 2) (43).

#### E3BP (Pdhx) deficiency

No animal model is reported for E3BP deficiency.

#### PDKs deficiency

There are only a few reported cases of PDK deficiency in children because there are four isoenzymes of PDKs that are variably distributed in mammalian tissues, and deficiency of one isoenzyme may be compensated by other isoenzymes. Additionally, PDK deficiency may result in an increase or decrease in ‘active’ PDC activity, causing some metabolic complications as discussed later. To better understand the importance of PDK isoenzymes in the regulation of PDC and hence glucose homeostasis under different metabolic states, deficiency of three isoenzymes of PDKs (PDK2, PDK3 and PDK4) by gene deletion have been created in mice and *C. elegans*. Phenotypically mutated mice look normal and as expected do not show any sign of PDC deficiency. Hence, these mouse models are primarily investigated for their impact on carbohydrate metabolism in tissues. We have discussed these models under the Tissue- or Cell-Specific PDC deficiency.

#### PDP1 deficiency

There is no animal model created for PDP1 deficiency, however, a spontaneous canine model of a null mutation in the *Pdp1* gene has been reported. It is presented here because it provides some interesting insights into this deficiency. Approximately 20% of the current Clumber and Sussex Spaniel population are carriers for a null mutation in the *Pdp1* gene. Homozygous dogs (*Pdp1^−/−^*) have increased levels of blood lactate and experience severe exercise intolerance due to minimal activation of PDC activity. Dietary therapy of L-carnitine and a high-fat diet were found to be beneficial (Table 2) (44).

## Tissue- or cell-specific PDC deficiency

Mouse models of PDH deficiency are employed to create tissue-specific PDC to investigate the importance of PDC in carbohydrate metabolism. These investigations are discussed below.

### Liver-specific PDH deficiency

A murine model of liver-specific PDC deficiency was developed by breeding *Pdha1^/flox8^* females with transgenic male mice harboring an *alb-Cre* gene (45). There was no embryonic lethality of male embryos in this breeding. Reduction in fat and lean body mass was observed in deficient mice. Liver slices from liver-specific-PDH-deficient male progeny showed no incorporation of [U-^14^C]-glucose into fatty acids but a compensatory increase in lipogenic capacity of epididymal adipose tissue from deficient mice was observed. Surprisingly, liver and peripheral insulin sensitivities were improved. Expression of several key genes in the lipogenic pathway and their regulators were downregulated in livers from PDH-deficient mice (Table 3) (46). The levels of mRNAs of several key glycolytic enzymes were increased, whereas the mRNA levels of hypoxia-induced factor-1α and PDK1 were decreased in livers from PDH-deficient male mice. These and other findings indicated that liver-specific PDC deficiency is sufficient to induce ‘aerobic glycolysis’ (aka Warburg effect) in the liver, although the mechanism(s) governing this change appears different from those that induce the Warburg effect in some cancer cells (Table 3) (47).

To evaluate the need of PDC-generated acetyl-CoA in metabolic processes during normal hepatocyte regeneration and malignant transformation in mice, Jackson et al. (48) found that glycolysis and the tricarboxylic acid cycle can be functionally dissociated by targeted PDC inactivation without impacting the normal proliferative capacity of normal hepatocytes and can modestly alter the growth of aggressive hepatoblastomas despite a marked reduction in acetyl-CoA and significant reductions in the tricarboxylic acid cycle intermediates. Interestingly, oxidative-phosphorylation activity was maintained in both the cell types by increasing mitochondrial mass to compensate for metabolic substrate abnormalities (Table 3).

### Pancreatic *β*-cell-specific PDH deficiency

To evaluate the role of PDC in glucose-stimulated insulin secretion, a β-cell-specific PDC deficient mouse model was created by breeding *Pdha1^flox8^* female mice with transgenic male mice carrying an *Ins-Cre* transgene (49). Pancreatic insulin content was decreased in 1-day-old male pups and remained at reduced levels in adulthood. Similarly, the plasma insulin levels were reduced and consequently blood glucose levels were elevated in affected male mice from the neonate period to adulthood. Furthermore, glucose tolerance was impaired in affected adult male mice (Table 3) (49, 50). Analyses of affected male mice revealed: (i) reduction in the number of islets per unit area, mean islet size, and the proportion of islet cells that were β-cells, (ii) reduction in the number of insulin-immunopositive extra-islet small endocrine cell clusters, and (iii) reduction in mRNA abundance of transcription factors responsible for β-cell lineage commitment, proliferation, and maturation, demonstrating that PDC also played the key role as a regulator of β-cell mass and plasticity (Table 3) (50). To investigate the effect of PDC deficiency on glucose-stimulated insulin secretion in adult mice, the *Pdha1* gene was deleted in the β-cells by tamoxifen-induced Cre expression in adult mice. The level of insulin secretion at 30 min after glucose injection was lower and the intraperitoneal glucose tolerance test was impaired in these mice. There was a corresponding compensatory enlargement of islets with clear internal vacuoles in *β-Pdha1KO* mice (Table 3) (51).

### Heart/skeletal muscle-specific and muscle-specific PDH deficiency

A heart/skeletal muscle-specific PDH-deficient mouse model was developed by breeding homozygous *Pdha1^flox8^* females with transgenic male mice harboring a *Mck-Cre* transgene. There was no embryonic lethality, and the progeny grew normally during the preweaning period. Upon weaning on a standard rodent diet on postnatal day 21, male progeny died around day 28; however, male progeny survived if weaned on a high-fat diet for at least 6 months (the end of the study). Heart PDC activity levels were less than 5% in males and about 50% in heterozygous females. Male mice developed left ventricular hypertrophy and reduced left ventricular systolic function. There was a significant increase in myocyte diameter. The cellular changes in the affected females were of lesser severity (Table 3) (52). Death of male progeny on a rodent diet (high in carbohydrate-derived calories) was primarily due to heart failure.

To study muscle-specific PDC deficiency, *Pdha1^flox8^* females were bred with transgenic males harboring a tamoxifen-inducible human skeletal actin promoter/Cre recombinase transgene (*iHSA-Cre*). Tamoxifen-induced PDH deletion had no impact on energy expenditure, muscle contractile function or muscle insulin sensitivity during low-intensity exercise performance in affected mice. However, high-intensity exercise capacity was severely impaired in skeletal-specific PDH-deficient mice, revealing the importance of PDC for glucose oxidation in skeletal muscle during high-intensity exercise (Table 3) (53).

### Heart-specific PDH deficiency

Inducible cardiac-specific PDH*α* knockout mice were generated by breeding floxed-females with transgenic male mice harboring the *α-MHC-CreER^T2^* transgene. PDH deficiency resulted in larger myocardial infarct size and macrophage infiltration in the hearts. Increased hypertrophy and fibrosis in deficient hearts were observed. There was impairment in glucose oxidation in deficient hearts that was not altered by dichloroacetate. Impairment in ischemic ATP-activated protein kinase and the phosphorylation of AMPK during ischemic/reperfusion were observed (Table 3) (54, 55).

### PDKs deficiency

Systemic PDK2 deficiency was created in mice by deletion of the *Pdk2* gene. PDH activation was lower in muscle from PDK2KO mice (PDK2^−/−^) than wild-type muscle, and total PDK activity was markedly lower in PDK2KO muscle. An increased PDK1 level in PDK2KO muscle compensated for the lack of PDK2, and this increase was correlated with higher levels of HIF-1α in the affected muscle (Table 3) (56), PDK2 deficiency in mice resulted in higher PDC activity and lower blood glucose levels in the fed, but not the fasting state. PDK4 deficiency caused similar effects only after fasting. Double deficiency (PDK2KO/PDK4KO) intensified these effects in both the fed and fasting states (Table 3) (57). Starvation decreased blood glucose more in homozygous PDK4-deficient mice than wild-type mice. ‘Active’ PDC was higher in several peripheral tissues except liver in starved PDK4KO mice vs. wild-type mice. A decreased rate of hepatic gluconeogenesis was consistent with lower levels of its precursors in the blood (Table 3) (58). In high-fat fed obese *Pdk4^−/−^* mice, fasting blood glucose levels were lower, glucose tolerance was slightly better, and insulin sensitivity was slightly greater compared with obese wild-type mice (Table 3) (59).

Estrogen deficiency causes an increase in the osteoclastic resorption of bone, and PDK2 activity is increased during osteoclast differentiation. PDK2 deficiency delayed bone loss and decreased the number of osteoclasts in ovariectomized mice (Table 3) (60).

Charcot–Marie-Tooth (CMT) neuropathy is a collection of hereditary motor and sensory disorders of the peripheral nerve form. An X-linked form of axonal CMT (CMTX6) is caused by a missense mutation (c. G473A; p. R158H) in the PDK3 gene in three generation kindred (61). Interestingly, this mutation binds with increased affinity to the lipoyl domain of the E2 in PDC (conferring hyperactivity of the mutant PDK3), causing hyperphosphorylation of PDC and its inactivation. Normally, a mutated PDK would be expected to increase PDC activity but this novel mutation with its increased binding affinity to the E2 inhibits PDC. This mutation resulted in decrease in energy metabolism and mitochondrial function in patient fibroblasts and iPSC-derived motor neurons (62). Three types of mutations were generated for analyses: (i) CRIPSR-Cas9 mediated knock-in a missense mutation at the conserved amino acid residue 159 (R159H) in the *Pdk2* gene in *C. elegans* (pdkh2R159H), and (ii) human wild-type (hPDK3WT) and mutant (hPDK3R158H) forms of PDK3 selectively expressed in GABAergic motor neurons in *C. elegans*. The findings from these mutants are summarized as: (i) reduction in body width, (ii) decreased ATP levels, (iii) increased susceptibility to oxidative stress, and (iv) impairment in axon-associated synaptic transmission. Additionally, mutant worms overexpressing human WT and p. R158H forms of PDK3 in the GABAergic motor neurons exhibited progressive neurodegeneration (62).

### E4F1 mutation causes Dlat deficiency

Although regulation of PDC activity is largely exerted by posttranslational modifications (primarily by phosphorylation/dephosphorylation), gene regulation at the transcriptional level also plays a significant role during early development, in some cancers and by hormonal status (1). The E4 transcription factor 1 (E4F1) regulates a set of genes [*Dlat*, *Dld*, *PDPR* (pyruvate dehydrogenase phosphatase 2 regulator), *Mpc1* (mitochondrial pyruvate carrier 1) and *Slc25a19* (solute carrier family 25 member 19)] involved in pyruvate oxidation (63). Employing Next Generation Sequencing technologies, Legati et al. identified two siblings with a homozygous missense mutation (E4F1^K144Q^) in the *E4F1* gene that were diagnosed with PDC deficiency (64). *E4F1* inactivation in E4F1^KO^ fibroblasts and E4F1 siRNA-treated cells exhibited a marked reduction in PDC activity and in pyruvate oxidation (63). Striated skeletal muscle-specific E4F1*^KO^* mice were generated by crossing E4F1^−/flox^ mice with *Acta-1-Cre* transgenic mice. These E4F1^KO^ mice developed hypercontracted fibers and centralized regenerative fibers in 18-momth-old animals. Expression of mRNA and protein levels of *Dlat* were markedly decreased and that of *Dld* to a lesser extent causing a marked reduction in PDC activity in E4F1^KO^ skeletal muscle. E4F1^KO^ mice exhibited lactic acidosis and a marked reduction in physical endurance. Feeding a ketogenic diet or administration of dichloroacetate to E4F1^KO^ mice were beneficial to improve lactic acidosis and physical endurance (63).

Mice lacking E4F1 in the central nervous system (CNS) were generated by crossing *E4F1* conditional KO mice with transgenic mice expressing the *Nes-Cre* transgene (65). E4F1 deficiency in the CNS resulted in perinatal death. E16.5 E4F1^(Nes)KO^ embryos exhibited degenerated areas in forebrain, midbrain and hindbrain neuroepithelium. Histological analyses revealed that the structural lesions were initiated at E14.5, increasing at later developmental stages. Analyses of mRNA and protein levels showed that the *Dlat* gene is highly dysregulated in the brain of E4F1^(Nes)KO^ embryos, causing reduction in PDC activity and the levels of acetyl-CoA. Circulating lactate levels were significantly increased in the mutant embryos compared to control embryos. Additional findings demonstrated that *E4F1* inactivation in the brain impacted activity of the Elongation complex (via its Elp3 subunit) and translation defects resulting from impaired tRNAs U_34_ modifications (65).

## Discussion

Animal models employing six different species express mutations in most of the genes responsible for encoding PDC proteins have been developed. Among these models, mouse models for the PDH*α* protein represent the largest number of investigations. This is not surprising, as the mutations in the *PDHA1* gene in patients account for over 80% of all mutations in the PDC genes (Table 1). Furthermore, mammalian PDC deficiency in mouse brain is of interest to investigate the cellular changes and molecular mechanisms affecting the brain in PDC-deficient patients. The other animal models are also of interest because they provide insights about mutations in the other PDC genes, and more importantly, they provide rapid drug screening platforms for PDC deficiency. There are several interesting findings from *Pdha1* deletion in mice. First, there is a threshold of reduction of approximately 25% of residual PDC activity for survival of the heterozygous female mice, as evident from the systemic-PDH-deficient female mice (17) and brain-specific PDH-deficient female mice (18). In the former study, nearly all tissues expressed Cre recombinase, and in the latter study Cre recombinase was expressed in the neurons and glia in the brain, indicating about 25% residual PDC activity can be tolerated for survival. In another study, in which *Pdha1* deletion was targeted in neurons and astrocytes, about 50% reduction in PDC activity in the brain was sufficient for male progeny to be born but it proved lethal by the postnatal day 28 (23). These observations are consistent with neonatal death of children with severe PDH deficiency.

Malformations of cerebral structures observed in the mice are like those reported for many PDC-deficient subjects with systemic PDH deficiency and brain-specific PDH deficiency, demonstrate the usefulness of the floxed^8^ mouse line generated by Johnson and associates (16). Additionally, these mouse models have provided new understanding about cellular proliferation, migration and differentiation of neurons in PDH-deficient mice during the prenatal and postnatal periods.

Furthermore, the metabolic flux studies using specifically radiolabeled glucose, acetate and propionate have documented the impairment in glucose-carbon flux into various metabolites in the tricarboxylic acid cycle and some amino acids and how the carbons from acetate and propionate supply two-carbon and four-carbon intermediates to preserve the neuron–glia metabolic interactions in PDH-deficient mice (22–24).

The mouse model for DLD deficiency has its inherent limitations because a homozygous state is embryonic lethal and the heterozygous state, with approximately one half of normal E3 activity, has no obvious phenotype reflecting no significant metabolic complications. Furthermore, severe E3 deficiency impacts the activities of other α-ketoacid dehydrogenase complexes, leading to a more complex clinical presentation. Interestingly, models for DLD deficiency in *C. elegans* and zebrafish appear to be useful for screening dietary and drug treatments, not only for DLD deficiency *per se* but also for PDC deficiency due to its component genes (if specific gene deletion models can be generated). The zebrafish and *Drosophila melanogaster* models for the *Pdhb* gene deletion are also useful for rapid screening for PDC deficiency and for investigating brain alterations associated with PDC dysfunctions. In contrast, mouse or any mammalian animal models for PDC deficiency are less suitable for primary screening of drugs for PDC deficiency (due to high cost and length of time requirement to perform screens) but are good models to perform secondary screens and to investigate the structural and functional impairment in the brain due to PDC deficiency.

### Genotype–phenotype association

Despite the availability of phenotypic information from several hundred PDC-deficient human subjects and identification of nearly 180 different mutations in the PDHA1 gene alone, no clear relationship has emerged about the genotype–phenotype association even for the most frequently occurring p. R263G mutation accounting for about 4% of all reported cases for PDHA deficiency (10). Attempts have been made for PDHA phenotypes (11) and even for the different genes of PDC and their clinical characteristics (66). The difficulty lies in there being considerable overlap of phenotypes within each gene and among other PDC genes. Some of the reason for variability in the phenotype among the patients with the same mutation could be the level of residual PDC activity, variation in X-inactivation in the different regions of the brain, how soon a diagnosis is made, and initiation of a high-fat diet as a treatment and the degree of restriction of carbohydrate in the diet. Animal models with a gene deletion (e.g., *Pdha1*) results in null mutation whereas most mutations in the human *PDHA1* gene are missense mutations expressing some residual PDC activity ranging from 5% to around 50% of the normal activity. This allows some carbon flux from pyruvate via PDC that could contribute to variability in the phenotype outcome. What is needed is creation of mouse models with specific point mutations (recreations of frequently reported missense mutations in the *PDHA1* gene) to evaluate a phenotypic expression with and without supplementation of a high-fat diet (very high fat and very low carbohydrate levels) to mothers from the initiation of lactation and to weaned pups from postnatal day 21 to evaluate the contribution of an alternate fuel to support developmental phenotype. Other animals reported in this review could be employed with specific point mutations in PDC genes to examine the genotype–phenotype relationship but their usefulness in understanding of cerebral structural and functional malformation likely would be very limited.

## Concluding remarks

Several animal models are reported for PDC genes, providing insights into malformation of brain structures and functions. These models have allowed investigators to evaluate the usefulness of a high fat diet and phenylbutyrate to provide alternate fuel and inhibition of PDKs, respectively. Since all reported mutations in the PDC genes in these models are null mutations, they resemble only a small number of PDC mutations in humans. It would be of interest to create point mutations especially in the *Pdha1* gene in mice and other animal species to better evaluate the possible genotype–phenotype relationship.
